# Inhibition of Acute Phase Inflammation by *Laminaria japonica* through Regulation of iNOS-NF-**κ**B Pathway

**DOI:** 10.1155/2013/439498

**Published:** 2013-10-31

**Authors:** Seong Kyu Park, Sook Jahr Park, Sang Mi Park, Il Je Cho, Chan Ik Park, Young Woo Kim, Sang Chan Kim

**Affiliations:** MRC-GHF, College of Oriental Medicine, Daegu Haany University, Gyeongsan 712-715, Republic of Korea

## Abstract

*Laminaria japonica* has been frequently used as food supplements in many of the Asian countries and as a drug in traditional oriental medicine. This research investigated the effects of *Laminaria japonica* extract (LJE) on acute phase inflammation in a carrageenan-induced paw edema model, as assessed by histomorphometric and immunohistochemical analyses. The effect of LJE was also evaluated in Raw264.7 cells stimulated with lipopolysaccharide (LPS) in the aspect of the inhibition of nitric oxide (NO), prostaglandin E_2_ (PGE_2_), and proinflammatory cytokines production. NO, PGE_2_, tumor necrosis factor (TNF)-**α**, interleukin-1**β**, and interleukin-6 contents were assayed by ELISA, and inducible NO synthase (iNOS) and cyclooxygenase (COX)-2 expressions were determined by western blot analyses. In rats, LJE treatment inhibited carrageenan-induced paw edema formation and infiltration of inflammatory cells in H&E staining. LJE treatment prevented the ability of LPS to increase the levels of iNOS and COX-2 protein in a concentration-dependent manner. Consistently, LJE suppressed the production of TNF-**α**, interleukin-1**β**, and interleukin-6. Treatment of the cells with LJE caused inhibition of inhibitor of **κ**B**α** phosphorylation induced by LPS, suggesting LJE repression of nuclear factor-**κ**B activity by LPS. In conclusion, this study shown here may be of help to understand the action mechanism of LJE and the anti-inflammatory use of *L. japonica*.

## 1. Introduction

Over the last few decades, natural products has been known to have beneficial effect to human beings. Some traditional food sources (especially seaweeds) contain high level of essentials for the body, and therefore, are able to maintain good heath by providing various nutrients [1]. *Laminaria japonica *is one of the most famous seaweeds called kombu (Japanese), dashima (Korean), or haidai (Chinese) and commonly consumed in Asian countries such as Republic of Korea, Japan, and China. In these countries, *L. japonica *is widely used as food supplements as well as a drug having effects on treating tumor, relieving phlegm, and regulating urine in traditional oriental medicine. Although it has been used for more than 1000 years as a drug in traditional medicine, the mechanism of beneficial effects was not clearly demonstrated.

Inflammation is a complex biological host response to harmful stimuli and is characterized by the classic signs such as redness, swelling, heat, and pain. A variety of studies has shown that a chronic or acute inflammatory state is closely associated with the pathogenesis of various disorders such as vascular disease, metabolic disease, obstructive pulmonary disease, infectious diseases, and cancer [2–6]. In these inflammatory processes, cytokines have important roles in progression of pathological states such as edema, intra-/intercellular stress, and tissue necrosis [7]. In particular, the proinflammatory cytokines such as tumor necrosis factor (TNF)-*α*, interleukin (IL)-1, and IL-6 and the pro-inflammatory enzymes including inducible nitric oxide synthase (iNOS) and cyclooxygenase (COX)-2 have been widely accepted with involvement of promoting inflammatory processes [8, 9].

In this study, we investigated the effects of the ethanol extract of *L. japonica* (LJE) as a novel anti-inflammatory candidate to inhibit paw edema formation in a rat model of acute inflammation. Furthermore, this study identifies LJE as a component with the inhibiting effects on the NO, prostaglandin E_2_ (PGE)_2_, and proinflammatory cytokines in macrophages treated with LPS. In terms of wide applications of *L. japonica* (i.e., food or drugs) and its therapeutic potential, the findings presented here demonstrate the important pharmacology of *L. japonica* and offer the possibility of its therapeutic applications for inflammatory diseases.

## 2. Materials and Methods

### 2.1. Preparation of the Ethanol Extract of *Laminaria japonica*

*L. japonica* was purchased from Daewon pharmacy (Daegu, Republic of Korea). The ethanol extract of *L. japonica* (LJE) was prepared by extracting 200 g of *L. japonica* in 1 L of 100% ethanol for 72 h. The ethanol extracts were filtered through a 0.2 *μ*m filter (Nalgene, New York, NY, USA), lyophilized, and stored at −20°C until use. The amount of LJE was estimated by the dried weight of lyophilized LJE. The yield of lyophilized LJE was 1.19%.

### 2.2. Analysis of LJE

LJE was analyzed by gas chromatography (GC) and mass spectrometry (MS) (Hewlett-Packard 6890N GC/MS, Palo Alto, CA, USA). The system is equipped with a HP-5MS column (30 m × 0.25 mm). The analyses were performed at 70°C (an initial temperature) and ramped with 2°C/min to 100°C (a final temperature) after equilibration. Injector temperature was 280°C. Carrier gas was helium with a flow of 1 mL/min. From the scanning data of the LJE profiling by GC and MS, we assessed the contents of the three marker components, palmitic acid, myristic acid, and oleic acid. For the analysis of oleic acid, the UPLC (ultra performance liquid chromatography) system (Waters, USA), which was equipped with a pump Waters ACQUITY ultra performance LC system (USA) and a Waters ACQUITY photodiode array detector (PDA), was used. The detection UV wavelength was set at 203 nm. For the palmitic acid and myristic acid, we used GC/MS (i.e., contents of three components in LJE were palmitic acid (89.35 *μ*g/mL); myristic acid (52.05 *μ*g/mL); oleic acid (13.8 *μ*g/mL)).

### 2.3. Materials

Horseradish peroxidase-conjugated goat anti-rabbit, anti-mouse, and anti-goat IgGs were purchased from KPL (Gaithersburg, MD, USA). Anti-phospho-I-*κ*B*α* and anti-COX-2 antibodies were supplied from Santa Cruz Biotechnology (Santa Cruz, CA, USA) and Cell signaling (Beverly, MA, USA), respectively. Antimurine iNOS antiserum was purchased from Transduction Laboratories (Lexington, KY, USA). Polyethylene glycol #400 (PEG) solution was obtained from yakuri Pure Chemical Co. (Kyoto, Japan). Carrageenan, dexamethasone, and other reagents were purchased from Sigma Chemical Co. (St. Louis, MO, USA).

### 2.4. Carrageenan-Induced Paw Edema

Animal studies were conducted in accordance with the institutional guidelines for care and use of laboratory animals [10]. Sprague-Dawley rats at 6 weeks of age (male, 140–160 g) were provided from Samtako Co. (Osan, Republic of Korea), acclimatized for 1 week, and maintained in a clean room at the Animal Center for Pharmaceutical Research, College of Oriental Medicine, Daegu Haany University. Animals were caged under the supply of filtered pathogen-free air, commercial rat chow (Purina, Republic of Korea), and water *ad libitum* at a temperature between 20 and 23°C with 12 h light and dark cycles and relative humidity of 50%. Rats (*N* = 24) were randomly divided into four groups, and thus, each group consisted of six animals. LJE, dissolved in 40% PEG, was orally administered to rats at the dose of 0.1 g or 0.3 g kg^−1^ day^−1^ for 3 consecutive days. Dexamethasone, an anti-inflammatory drug, was used as a positive control [11]. To induce acute phase inflammation in paw, rats were injected subcutaneously into the hind paw with a 1% solution of carrageenan dissolved in saline after vehicle or LJE treatment. The paw volumes were measured up to 4 h after the injection at intervals of 1 h. The hind paw volume was determined volumetrically by measuring with a plethysmometer (Letica, Rochester, MI, USA).

### 2.5. Histological Process

The hind paw skins—*dorsum* and *ventrum pedis* skins—were separated and fixed in 10% neutral buffered formalin, then, embedded in paraffin, sectioned (3~4 *μ*m), and stained with hematoxylin and eosin (H&E), and after that the histopathological profiles of each sample were observed under light microscope (Nikon, Japan) [12].

### 2.6. Histomorphometry

The thicknesses of *dorsum pedis* and *ventrum pedis* skins (from epidermis to dermis; keratin layers were excluded) were measured using automated image analyzer (DMI-300 Image Processing; DMI, Korea) under magnification 40 of microscopy (Nikon, Japan) at prepared skin histological samples as mm paw^−1^, and the infiltrated inflammatory cells were also counted using automated image analyzer as cells mm^−2^ of histological fields under magnification 200 of microscopy according to Kim et al. (2006) and some modifications [12].

### 2.7. Cell Culture

Raw264.7 cell, a murine macrophage cell line, was obtained from American Type Culture Collection (Rockville, MD, USA). The cells were maintained in Dulbecco's modified Eagle's medium (DMEM) containing 10% fetal bovine serum (FBS), 50 U mL^−1^ penicillin, and 50 *μ*g mL^−1^ streptomycin at 37°C in a humidified atmosphere with 5% CO_2_. For all experiments, the cells were grown to 80–90% confluency and were subjected to no more than 20 cell passages. Raw264.7 cells were incubated with 1 *μ*g mL^−1^ LPS (*Escherichia coli* 026:B6; Sigma, St. Louis, MO, USA). The cells were incubated in the medium without 10% FBS for 12 h and then exposed to LPS or LPS + LJE for the indicated time periods. LJE being dissolved in dimethylsulfoxide was added to the incubation medium 1 h prior to the addition of LPS.

### 2.8. MTT Cell Viability Assay

The cells were plated at a density of 5 × 10^4^ cells per well in a 96-well plate to determine any potential cytotoxicity [13]. Cells were serum-starved for 12 h and then were treated with LJE for the next 24 h.

### 2.9. Assay of Nitrite Production

NO production was monitored by measuring the nitrite content in culture media. This was performed by mixing the samples with Griess reagent (1% sulfanilamide, 0.1% *N*-1-naphthylenediamine dihydrochloride, and 2.5% phosphoric acid). Absorbance was measured at 540 nm after incubation for 10 min.

### 2.10. Immunoblot Analysis

Cells were lysed in the buffer containing 20 mM Tris·HCl (pH 7.5), 1% Triton X-100, 137 mM sodium chloride, 10% glycerol, 2 mM EDTA, 1 mM sodium orthovanadate, 25 mM *β*-glycerophosphate, 2 mM sodium pyrophosphate, 1 mM phenylmethylsulfonylfluoride, and 1 *μ*g mL^−1^ leupeptin [14]. Proteins of interest were visualized using ECL chemiluminescence detection kit. Equal loading of proteins was verified by actin immunoblottings. At least three separate experiments were performed to confirm changes.

### 2.11. Enzyme-Linked Immunosorbent Assay (ELISA)

Raw264.7 cells were preincubated with LJE for 1 h and continuously incubated with LPS for 24 h. Prostaglandin E_2_ (PGE_2_), TNF-*α*, IL-1*β*, and IL-6 contents in the culture media were measured by ELISA using anti-mouse PGE_2_, TNF-*α*, IL-1*β*, or IL-6 antibodies and biotinylated secondary antibody according to the manufacturer's instruction (Endogen, Woburn, MA, USA).

### 2.12. Scanning Densitometry

Scanning densitometry of the immunoblots was performed with an Image Scan & Analysis System (Alpha-Innotech, San Leandro, CA, USA). The area of each lane was integrated using the software AlphaEase version 5.5 (Alpha-Innotech) followed by background subtraction.

### 2.13. Statistical Analysis

For animal experiments, multiple comparison tests for different dose groups were conducted. Variance homogeneity was examined using the Levene test. If the Levene test indicated no significant deviations from variance homogeneity, the obtained data were analyzed by one way ANOVA test followed least significant difference (LSD) test, Kruskal-Wallis *H* test or the Mann-Whitney *U* (MW) test. Statistical analyses were conducted using SPSS for Windows (Release 14.0K, SPSS Inc., USA). Differences were considered significant at *P* < 0.05. In addition, the changes between carageenan control and test material administered groups were also calculated to help the understanding of the efficacy of test material as follows: Percentage Changes Compared with carageenan Control (%) = ((Data of test material treated groups − Data of carageenan control)/(Data of carageenan control) × 100). For cell experiments, one-way analysis of variance (ANOVA) was used to assess statistical significance of differences among treatment groups. For each statistically significant effect of treatment, the Newman-Keuls test was used for comparisons between multiple group means. The data were expressed as means ± 95% confidence intervals (CI). All statistical tests were two-sided.

## 3. Results

### 3.1. Inhibitory Effect of LJE on Carrageenan-Induced Paw Edema

To determine inhibitory effects of LJE on acute inflammation, we used the carrageenan-induced paw edema model because this model is widely employed for screening the effects of anti-inflammatory drugs [15]. 

**Figure 1:**
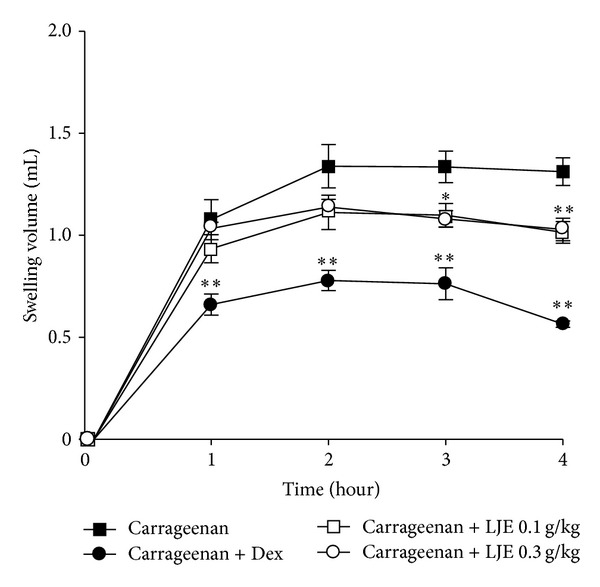
Inhibition of carrageenan-induced paw edema formation by the ethanol extract of* Laminaria japonica *(LJE). LJE was administered for 3 days to rats at the oral dose of 0.1 or 0.3 g kg^−1^ day^−1^ prior to the induction of paw edema. Paw edema was induced by subcutaneously injecting a 1% solution of carrageenan dissolved in saline (0.1 mL per animal) into the hind paw. The thickness of the paw was measured before and 1–4 h after carrageenan injection. Data point represents the swelling volume of the paw, which was standardized with the thickness of the paw volume before carrageenan injection. Dexamethasone (Dex, 1 mg kg^−1^, p.o.) was used as a positive control. Data represents the mean ± S.E.M. of six animals (significant as compared with carrageenan alone, *P < 0.05, **P < 0.01).

Because it has been shown that the injection of carrageenan induces acute inflammatory responses including paw swelling and increases in neutrophil infiltration, we focused in this study on the anti-inflammatory effects of LJE in the paw injected with carrageenan. We found that paw edema formation began to be observed as early as 1 h after a carrageenan injection and that the edema induction reached a maximum at 2 h after treatment (Figure 1). Inflammatory paw swellings persisted at least up to 4 h after treatment. Oral administration of LJE at the dose of 0.1 or 0.3 g kg^−1^ day^−1^ to rats significantly blocked the carrageenan-induced paw swellings. We confirmed that dexamethasone treatment (1 mg kg^−1^ day^−1^, p.o.), a positive control, also effectively decreased edema formation. 

### 3.2. Inhibitory Effect of LJE on Acute Inflammation in Rats

**Figure 2:**
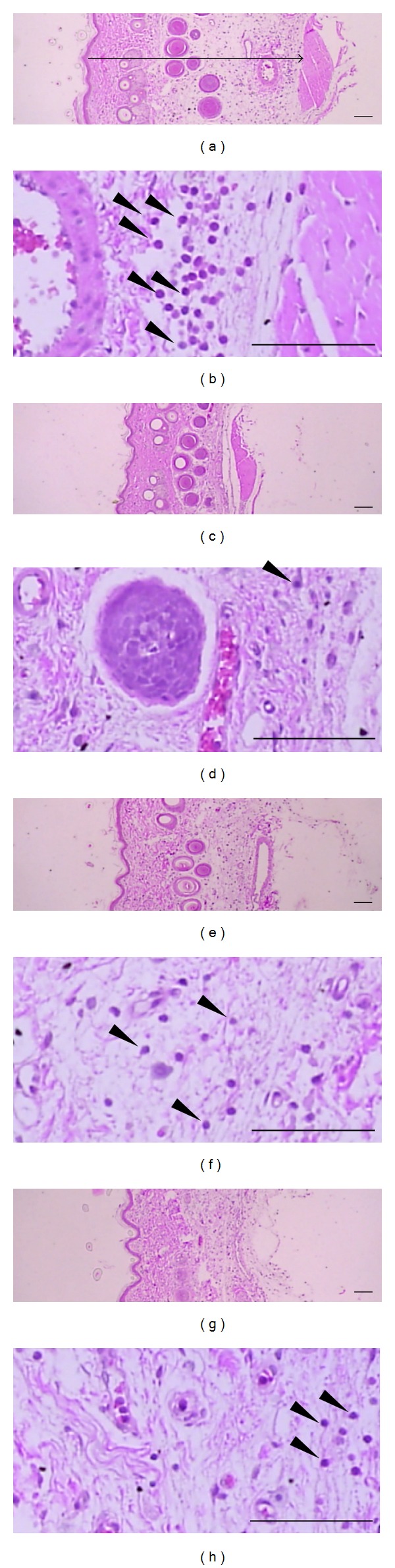
Changes in histological profiles of the *dorsum pedis* skin in carrageenan control (a, b), dexamethasone (c, d), LJE 0.1 g kg^−1^ day^−1^ (e, f), and LJE 0.3 g kg^−1^ day^−1^ (g, h) treated groups. Note that marked increases of skin thicknesses due to edematous changes were detected by carrageenan treatment with increases of inflammatory cell infiltrations. However, these increases of skin thicknesses and inflammatory cell infiltrations were effectively inhibited by treatment with dexamethasone and with two different dosages of LJE 0.1 g kg^−1^ day^−1^ and 0.3 g kg^−1^ day^−1^, respectively. Arrow indicates total thicknesses measured. Arrow heads showed infiltrated inflammatory cells. All H&E stain; scale bars = 160 *μ*m.

**Figure 3:**
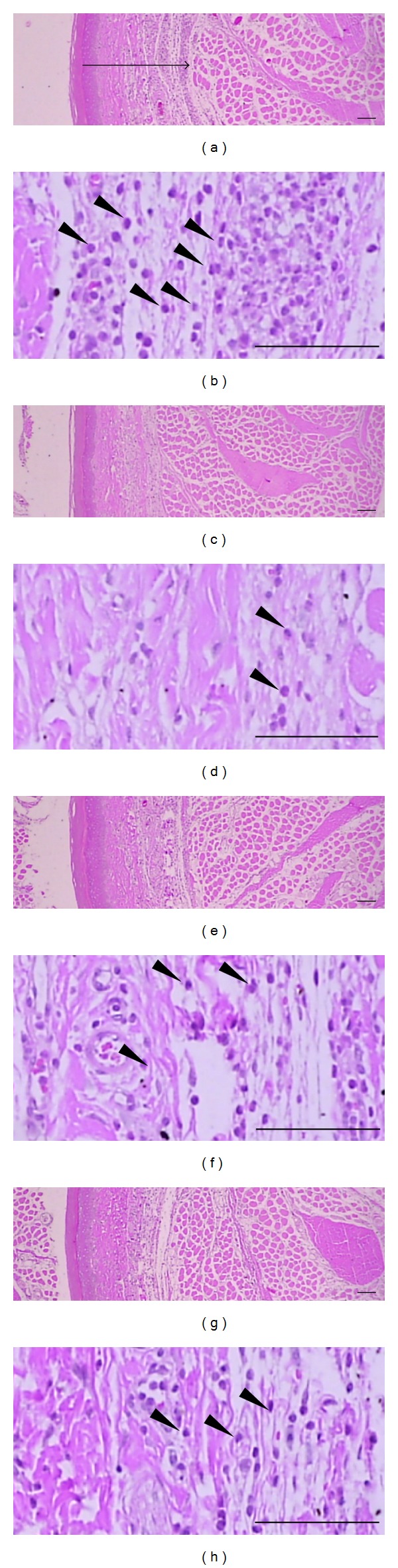
Changes in histological profiles of the *ventrum pedis* skin in carrageenan control (a, b), dexamethasone (c, d), LJE 0.1 g kg^−1^ day^−1^ (e, f), and LJE 0.3 g kg^−1^ day^−1^ (g, h) treated groups. Note that marked increases of skin thicknesses due to edematous changes were detected by carrageenan treatment with increases of inflammatory cell infiltrations quite similar to those of *dorsum pedis* skins. However, these increases of skin thicknesses and inflammatory cell infiltrations were effectively inhibited by treatment with dexamethasone and with two different dosages of LJE 0.1 g kg^−1^ day^−1^ and 0.3 g kg^−1^ day^−1^, respectively. Arrow indicates total thicknesses measured. Arrow heads showed infiltrated inflammatory cells. All H&E stain; scale bars = 160 *μ*m.

In addition, we determined the effects of LJE on histological profiles of *dorsum pedis* and the *ventrum pedis* skin (Figures 2 and 3). The histomorphometrical measurements of the *dorsum pedis* and *ventrum pedis* skin were listed in Table 1. In the present study, marked increases of infiltrated inflammatory cells and of skin thicknesses on both *dorsum* and *ventrum pedis* were detected by treatment of carrageenan (Figures 2 and 3). However, these carrageenan-induced acute edematous inflammatory changes were significantly (*P* < 0.01) inhibited by treatment with both different dosages of LJE and dexamethasone (Table 1). The thickness of *dorsum pedis* skin in dexamethasone and LJE 0.1 and 0.3 g kg^−1^ day^−1^ treated groups was changed as −55.74, −26.07, and −34.24% as compared with carrageenan control, respectively. The thickness of *ventrum pedis* skin in dexamethasone and LJE 0.1 and 0.3 g kg^−1^ day^−1^ treated groups was changed as −30.28, −11.19, and −22.28% as compared with carrageenan control, respectively. The number of infiltrated inflammatory cells on the *dorsum pedis* skin in dexamethasone and LJE 0.1 and 0.3 g kg^−1^ day^−1^ treated groups was changed as −79.73, −47.30, and −54.05% as compared with carrageenan control, respectively. The number of infiltrated inflammatory cells on the *ventrum pedis* skin in dexamethasone and LJE 0.1 and 0.3 g kg^−1^ day^−1^ treated groups was changed as −92.45, −68.09, and −88.81% as compared with carrageenan control, respectively. These results suggest that LJE prevents the acute phase of swelling implicated with inflammation *in vivo*.

### 3.3. Inhibition Effect of LJE on NO and PGE_2_ Induced by LPS Treatment

**Figure 4:**
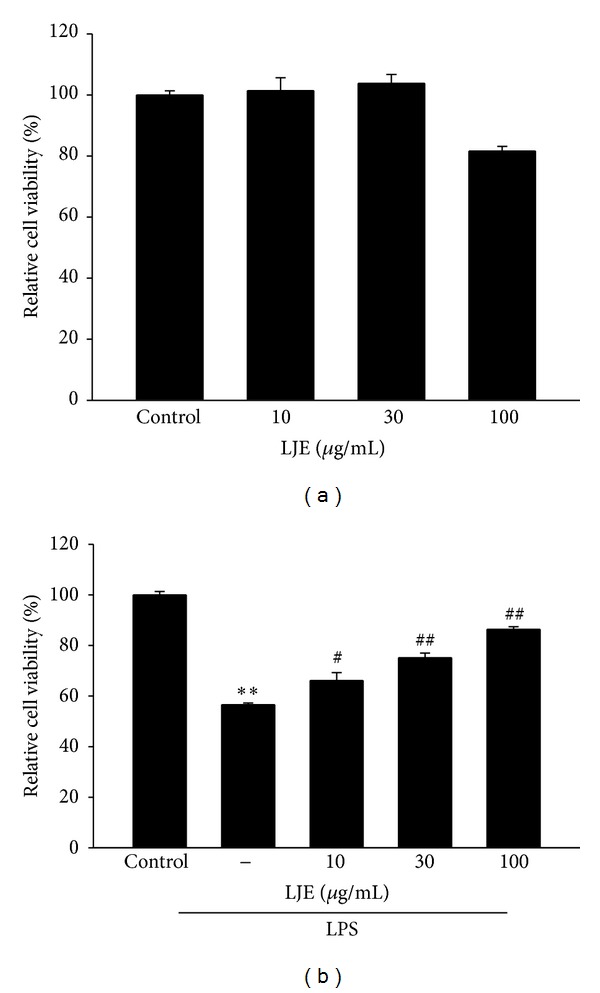
The inhibitory effects of LJE on cell viability. (a) MTT assay. Raw264.7 cells were treated with 10, 30, or 100 *μ*g mL^−1^ LJE. (b) MTT assay. Raw264.7 cells were treated with 10, 30, or 100 *μ*g mL^−1^ LJE for 1 h and continuously incubated with LPS (1 *μ*g mL^−1^) for 24 h. Data represents the mean ± S.E.M. from three separate experiments (significant as compared with vehicle-treated control, **P < 0.01; significant as compared with LPS alone, ^#^ *P* < 0.05, ^##^ *P* < 0.01).

**Figure 5:**
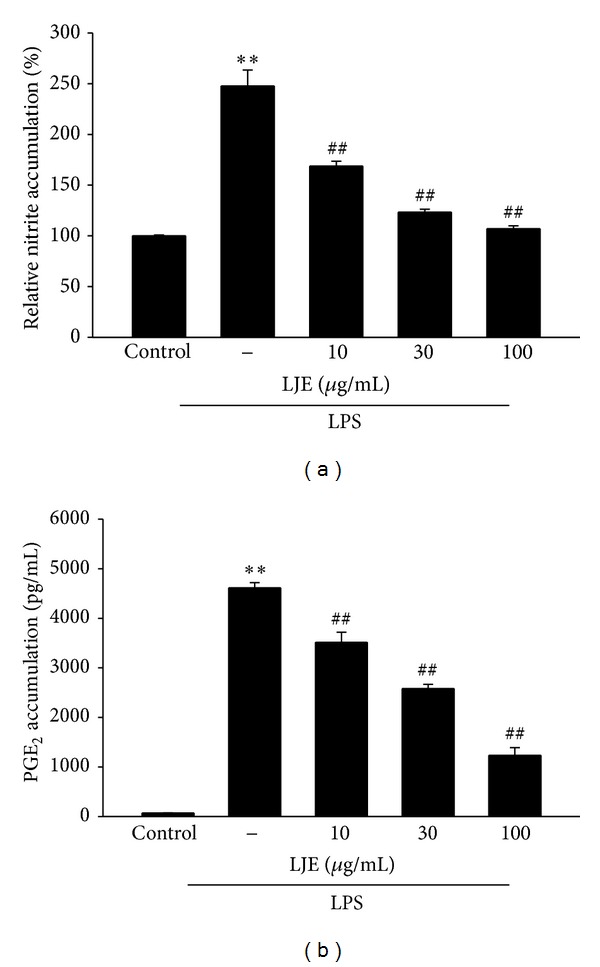
The inhibitory effects of LJE on nitric oxide (NO) and prostaglandin E_2_ (PGE_2_) induction by LPS. (a) NO and (b) PGE_2_ production. Raw264.7 cells were treated with 10, 30, or 100 *μ*g mL^−1^ LJE for 1 h and continuously incubated with LPS (1 *μ*g mL^−1^) for the next 24 h. NO and PGE_2_ concentrations in culture media were monitored, as described in Section 2. Data represents the mean ± S.E.M. from three separate experiments (significant as compared with vehicle-treated control, **P < 0.01; significant as compared with LPS alone, ^##^ *P* < 0.01).

Next, we verified the anti-inflammatory effect of LJE *in vitro*. We determined any possible toxicity of LJE in Raw264.7 cells. MTT assay indicated that cell viability was not affected by LJE treatment up to 100 *μ*g mL^−1^ (Figure 4(a)). Moreover, LJE restored LPS-inducible cell toxicity (Figure 4(b)). 10–100 *μ*g mL^−1^ concentrations of LJE were chosen to determine the effect of LJE on NO and PGE_2_ production in Raw264.7 cells. Production of the NO and PGE_2_ was measured in the media of Raw264.7 cells treated with LPS with or without LJE as described in Section 2. LPS treatment (1 *μ*g mL^−1^, for 24 h) increased NO production by 2.5-fold, which was markedly deceased by concomitant treatment with LJE in a concentration-dependent manner (Figure 5(a)). In addition, LJE treatment significantly inhibited PGE_2_ production in Raw264.7 cell treated with LPS (Figure 5(b)).

### 3.4. Inhibition of LPS-Inducible TNF-*α*, IL-1*β*, and IL-6 Productions by LJE Treatment

**Figure 6:**
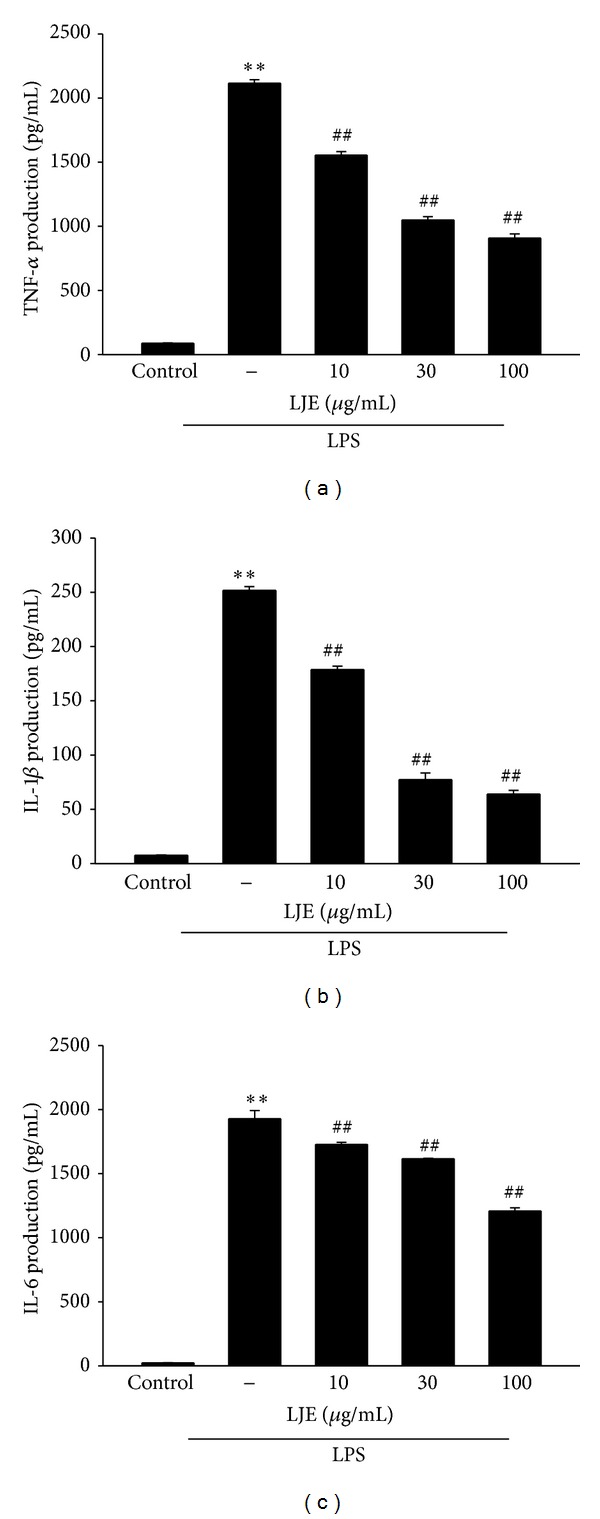
The inhibitory effects of LJE on TNF-*α*, IL-1*β*, and IL-6 production by LPS. (a) TNF-*α*, (b) IL-1*β*, and (c) IL-6 contents in culture media. Raw264.7 cells were treated with 10, 30, or 100 *μ*g mL^−1^ LJE for 1 h and continuously incubated with LPS (1 *μ*g mL^−1^) for 24 h. Data represents the mean ± S.E.M. from three separate experiments (significant as compared with vehicle-treated control, **P < 0.01; significant as compared with LPS alone, ^##^ *P* < 0.01).

Next, we assessed the effects of LJE on pro-inflammatory cytokines such as TNF-*α*, IL-1*β*, and IL-6. Production of the cytokines was measured by using ELISA assays in the media of Raw264.7 cells stimulated by LPS (1 *μ*g mL^−1^) alone or in combination with LJE. Treatment of the cells with LPS increased the production of the cytokines (Figures 6(a)–6(c)). LJE treatment effectively decreased the production of the cytokines, indicating that the anti-inflammatory effects of LJE might, at least in part, be related to LJE inhibition of LPS-inducible pro-inflammatory cytokines productions. 

### 3.5. Effect of LJE on LPS-Inducible iNOS and COX-2 Expression

**Figure 7:**
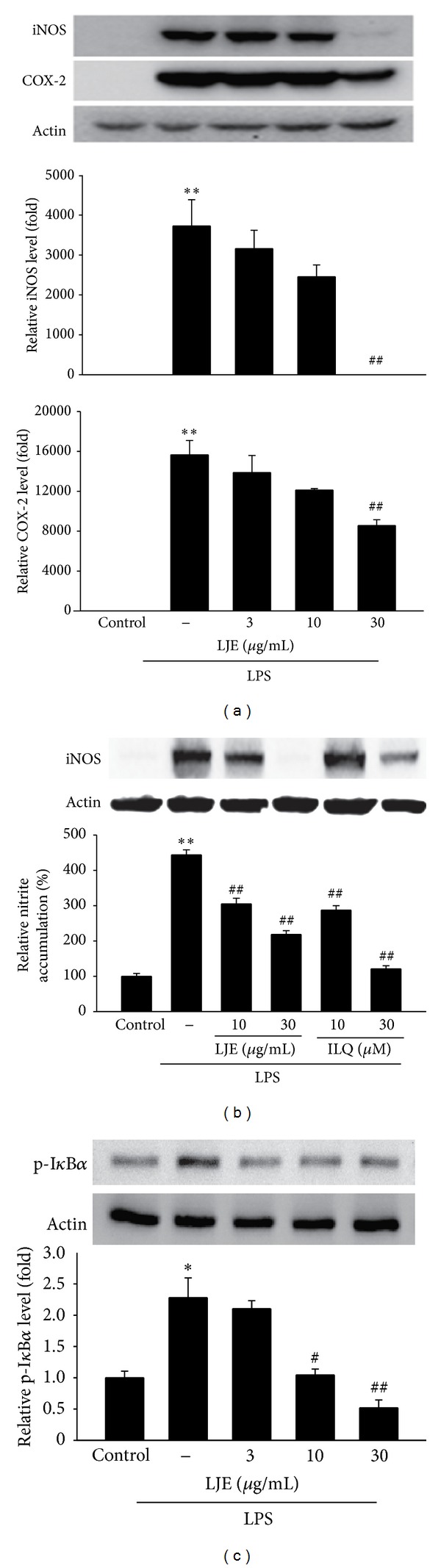
Inhibition of LPS-induced iNOS and COX-2 expression by LJE. (a) Immunoblottings for iNOS and COX-2. iNOS or COX-2 protein levels were monitored 12 h after treatment with LPS (1 *μ*g mL^−1^) alone or in combination with LJE (3, 10, or 30 *μ*g mL^−1^). (b) Inhibition of LPS-induced iNOS expression and NO induction by LJE and isoliquiritigenin (ILQ). iNOS protein levels and NO concentration were monitored as described in legends of Figures 7(a) and 5(a), respectively. (c) Immunoblottings for phosphorylated I-*κ*B*α* (p-I-*κ*B*α*). The cells were treated with LPS or LPS + LJE for 30 min. The relative protein levels were measured by scanning densitometry. For a, b, and c, data represents the mean ± S.E.M. from three separate experiments (significant as compared with vehicle-treated control, *P < 0.05, **P < 0.01; significant as compared with LPS alone, ^#^ *P* < 0.05, ^##^ *P* < 0.01).

iNOS and COX-2 are key enzymes in the aspect of the induction of proinflammatory cytokines. NF-*κ*B is the important transcription factor for the inflammatory genes such as iNOS and COX-2 and is translocated to the nucleus by phosphorylation of I-*κ*B*α* and subsequent proteolytic degradation of I-*κ*B*α* subunit [16–19]. We then assessed the protein expression of iNOS and COX-2 by western blotting. Although treatment with LPS significantly induced iNOS and COX-2 expression, LJE treatment (3–30 *μ*g mL^−1^) prevented the iNOS and COX-2 induction (Figure 7(a)). In particular, LJE treatment at 30 *μ*g mL^−1^ almost completely decreased induction of iNOS expression by LPS, similar to the result using isoliquiritigenin (a known anti-inflammatory flavonoid in licorice) (Figure 7(b)). Moreover, LPS exposure increased phosphorylation of I-*κ*B*α*, and LJE inhibited LPS-inducible I-*κ*B*α* phosphorylation (Figure 7(c)). Thus, LJE might prevent iNOS and COX-2 gene induction by inhibiting I-*κ*B*α* phosphorylation. 

## 4. Discussion

Studies have shown that the extracts of *L. japonica *have multiple bioactivities such as antiallergenic, antioxidant, and anticoagulant effects [20–23]. It has been shown that its ethanol extract has antiallergenic effects and the lipophilic extracts from *L. japonica* have protective effects against oxidant stress [20, 21]. The fucoidan fractions from *L. japonica* have anticoagulant potentials, and the hydrolyzed oligosaccharides from water extract are involved in antiapoptotic activity in mouse thymocytes [22, 23]. Moreover, it is well established that inflammatory and oxidative stress has been implicated in the important factor for the onset of various diseases. Moreover, the pathological process of inflammation may be associated with oxidative stress and the production of free radicals. Therefore, we investigated the effects of LJE on acute inflammation. In this study, we used two different approaches: (1) *in vivo* studies involving paw edema model in rats injected with carrageenan and (2) *in vitro* studies using Raw264.7 murine macrophage treated with LPS.

Swelling is one of the most important symptoms of acute inflammation, which is characterized by an increase in vascular permeability and infiltration of cells. Carrageenan-induced paw edema is commonly used for screening of new anti-inflammatory drug candidates and is a well-established rat model of edema [10]. The intraplantar injection of carrageenan induces inflammatory responses (i.e., increases in paw edema and neutrophil infiltration and development of hyperalgesia) [15]. At the site of inflammation, cytokines have been studied to have an important role in formation of edema by inducing vasodilatation and increasing of vascular permeability [24]. In particular, recent studies have shown that carrageenan induces peripheral release of NO as well as PGE_2_ [25]. In addition, it has been shown that carrageenan also induces the release of TNF-*α*, which subsequently promotes IL-1 and IL-6 production in the tissue [26]. 

Our carrageenan-induced rat paw edema model enabled us to demonstrate the ability of LJE to inhibit paw edema in rats injected with carrageenan. Carrageenan successfully induced paw swelling by subcutaneous injection. As demonstrated in the present study, treatment with LJE significantly decreased the induction of paw edema. In histological analysis, we verified the inhibitory effects of LJE on acute inflammation in rats. In hematoxylin and eosin stating, LJE markedly inhibited skin thicknesses on both *dorsum* and *ventrum pedis* induced by carrageenan. Moreover, administration of LJE also inhibited infiltration of inflammatory cells increased by carrageenan injection. This result demonstrates that LJE could inhibit the acute inflammation in rats.

Macrophages play crucial role in immune defense mechanism during infection and in the process of development of human diseases. LPS, a prototypical endotoxin, can directly activate macrophages, which can produce inflammatory mediators [27–29]. Thus, pharmacological intervention of LPS-inducible inflammatory mediators (e.g., NO, TNF-*α*, and ILs) is thought as one of the most important strategies to treat various diseases caused by activation of macrophages. 

The inflammatory response is mediated by various signaling molecules and enzymatic pathways, among which iNOS and COX-2 are essential enzymes playing an important role in regulating the formation of NO and PGs during inflammation, respectively [6, 7, 24]. Importantly, it has been shown that macrophages from iNOS knock-out mice are protected from acute inflammation induced by LPS/IFN*γ* [30]. Therefore, we tested in this study whether LJE decreased the level of LPS-induced NO and PG production. Treatment with LJE markedly deceased NO and PGE_2_ production in Raw264.7 cell treated with LPS. Moreover, immunoblot analyses revealed that LJE effectively blocked the induction of iNOS and COX-2 proteins. The iNOS and COX-2 promoter comprises the binding sites for transcription factors such as NF-*κ*B and AP-1 [31]. In particular, NF-*κ*B is an essential transcription factor necessary for the iNOS and COX-2 gene transcription.

The pro-inflammatory cytokines such as TNF-*α*, IL-1, and IL-6 are small secreted proteins which mediate and regulate immunity and inflammation. TNF-*α* is a major mediator in inflammatory responses and can induce innate immune responses by activating macrophages and by stimulating secretion of other inflammatory cytokines [32]. Therefore, TNF-*α* is thought to be a principal mediator in LPS-inducible tissue injury [33]. IL-1*β* is another inflammatory cytokine, which is found in the circulation following Gram-negative sepsis, and IL-6 is also an inflammatory cytokine mainly synthesized by macrophages and plays a role in the acute phase response [34, 35]. Here, we evaluated the inhibitory effects of LJE on cytokines in LPS-stimulated macrophages. Our results indicated that LJE significantly inhibited LPS-induced TNF-*α*, IL-1*β*, and IL-6 secretions. In addition, it has been reported that carrageenan induces the release of NO, PGE_2_, and TNF-*α* production in the tissue [26]. Our carrageenan-induced rat paw edema model enabled us to demonstrate the ability of LJE to inhibit edema induced by acute inflammation. LJE administration notably decreased the paw edema induction. These results in conjunction with the marked inhibition of LPS-induced NO, PG, and TNF-*α* productions by LJE in macrophages imply that the antiedematous effects of LJE might result from its inhibition of NO and TNF-*α* synthesis in the peripheral tissues.

NF-*κ*B, the transcription factor, participates in the regulation of various genes involved in the process of inflammatory and immune responses, cell adhesion, and survival [17–19]. NF-*κ*B activation mediates transactivation of iNOS and COX-2 and many pro-inflammatory genes including TNF-*α*, IL-1, IL-6, and IL-8 [34–38]. It is well recognized that many anti-inflammatory drugs have inhibitory effects on cytokines by inhibiting NF-*κ*B activation [39]. NF-*κ*B is regulated by its interaction with I-*κ*B*α*, its inhibitor protein. NF-*κ*B became activated by degradation of I-*κ*B*α* following I-*κ*B*α* phosphorylation, and then, the activated NF-*κ*B translocates into the nucleus. Our results showing that LPS caused the phosphorylation of I-*κ*B*α*, whereas pretreatment with LJE significantly inhibited the process, suggest that LJE might inhibit NF-*κ*B activation due to its inhibition of I-*κ*B*α* phosphorylation.

Our research results demonstrate that LJE was found to have an anti- inflammatory effect in acute inflammation model using the carrageenan-induced paw edema model *in vivo*. More importantly, LJE exerts anti-inflammatory effects *in vitro*, which results from the inhibition of NF-*κ*B activation in macrophages, thereby, inhibiting the production of iNOS and COX-2 and proinflammatory cytokines. Moreover, we also verified the three main components in LJE including palmitic acid, myristic acid, and oleic acid. Previous studies have shown that various fatty acids have beneficial effects, although each part of LJE has different amount of fatty acid [40, 41]. Therefore, it remains to confirm the effects of three components in LJE on acute phase of inflammation and what is the active compound in LJE. In conclusion, our findings showing the inhibition of paw edema by LJE as well as inflammatory gene induction may be of help to understand the pharmacology and action mechanism of LJE. Although its effects on acute inflammation might be preventive in this study, we showed here the existence of a new candidate for anti-inflammatory herb in terms of therapeutic potential.

## Figures and Tables

**Table 1:** Changes on the histomorphometrical analysis of hind paw skins in the present study (group summary).

Groups	*Dorsum pedis* skin		*Ventrum pedis* skin	
	Thickness (epidermis to dermis; mm)	Infiltrated inflammatory cells (cells/mm^2^ of cutaneous regions)	Thickness (mm)	Infiltrated inflammatory cells (cells/mm^2^ of cutaneous regions)
Controls				
Carrageenan	2.424 ± 0.471	118.40 ± 55.25	1.133 ± 0.078	1358.80 ± 332.34
Reference				
Dexamethasone	1.073 ± 0.144^a^	24.00 ± 6.96^c^	0.790 ± 0.136^a^	102.60 ± 38.33^c^
LJE treated as				
0.1	1.792 ± 0.239^a^	62.40 ± 11.15^d^	1.006 ± 0.048^b^	433.60 ± 355.78^d^
0.3	1.594 ± 0.254^a^	54.40 ± 3.98^c^	0.880 ± 0.064^a^	152.00 ± 67.31^c^

Values are expressed as mean ± SD of 5 rat's hind paws.

^a^ *P* < 0.01 and ^b^ *P* < 0.05 compared to carrageenan control by LSD test.

^c^ *P* < 0.01 and ^d^ *P* < 0.05 compared to carrageenan control by MW test.

## Abbreviations

I-*κ*B: Inhibitor of *κ*B
IL: Interleukin
iNOS: Inducible nitric oxide synthase
LJE: *Laminaria japonica* extract
LPS: Lipopolysaccharide
NF-*κ*B: Nuclear factor-*κ*B
NO: Nitric oxide
PGE_2_: Prostaglandin E_2_
TNF-*α*: Tumor necrosis factor-*α*.
